# Balancing policy ambition and systems reality in expanding access to non-communicable diseases medicines: lessons from Ghana and Georgia

**DOI:** 10.1093/heapol/czag082

**Published:** 2026-06-25

**Authors:** Augustina Koduah, Akaki Zoidze, Maia Uchaneishvili, Jacob Novignon, Adrianna Murphy, Benjamin Palafox, Tolib Mirzoev, George Gotsadze

**Affiliations:** Department of Pharmacy Practice and Clinical Pharmacy, School of Pharmacy, University of Ghana, P.O. Box LG 43, Legon, Ghana; Curatio International Foundation, 3 Kavsadze str, Office 5, Tbilisi 0179, Georgia; School of Natural Sciences and Medicine, Ilia State University, Kakutsa Cholokashvili Ave 3/5, Tbilisi 0179, Georgia; Curatio International Foundation, 3 Kavsadze str, Office 5, Tbilisi 0179, Georgia; Centre for Social Policy Studies, University of Ghana, P.O. Box LG 72, Legon, Ghana; Department of Health Services Research and Policy, London School of Hygiene & Tropical Medicine, 15-17 Tavistock Place, London WC1H 9SH, United Kingdom; Department of Global Health and Development, London School of Hygiene & Tropical Medicine, 15-17 Tavistock Place, London WC1H 9SH, United Kingdom; Department of Global Health and Development, London School of Hygiene & Tropical Medicine, 15-17 Tavistock Place, London WC1H 9SH, United Kingdom; Curatio International Foundation, 3 Kavsadze str, Office 5, Tbilisi 0179, Georgia; School of Natural Sciences and Medicine, Ilia State University, Kakutsa Cholokashvili Ave 3/5, Tbilisi 0179, Georgia

**Keywords:** access to medicines, financing medicines, Ghana, Georgia, NCDs

## Abstract

Ensuring sustained access to affordable, high-quality medicines is central to achieving universal health coverage (UHC) in low- and middle-income countries (LMICs). This is particularly true for chronic non-communicable diseases (NCDs), which require long-term, uninterrupted treatment. Although global guidance recommends expanding pharmaceutical benefits, regulating prices, promoting generics, and strengthening procurement and supply chains, many LMICs continue to face inequities in medicine availability and high out-of-pocket costs. The experiences of Ghana and Georgia, two countries that have invested heavily in UHC reforms, pharmaceutical benefits, centralized procurement, and pricing policies, illustrate gaps between policy ambitions and implementation realities in expanding access to NCD medicines. This paper examines four health-system factors influencing access to NCD medicines: system capacity, provider behaviour, digital enablers, and public trust. In both countries, centralized procurement faces forecasting inaccuracies, procurement delays, supply chain weaknesses, and distribution challenges, leading to stockouts and inconsistent availability. Provider behaviours further influenced access, with reimbursement delays, low payment rates, and administrative burdens contributing to informal fees, off-list prescribing, and suboptimal use of generics. Investments in digital systems such as logistics management information systems and electronic prescribing showed promise but were limited by uneven adoption, data quality issues, and insufficient integration into decision-making processes. Public awareness and trust emerged as critical yet neglected determinants. Limited communication and perceptions of low quality of state-purchased medicines reduced uptake of benefits in Georgia, while gaps between National Health Insurance Scheme (NHIS) intentions and patient experiences constrained equitable access in Ghana. Strengthening access to NCD medicines requires system-wide readiness, aligned provider-patient incentives, effective digital integration, and sustained community engagement to build trust and support uptake.

Key messages

- Expanding affordable access to NCD medicines requires effective implementation of pharmaceutical policies within well-functioning health systems.
- In Ghana and Georgia, the implementation of well-designed policies was limited by bottlenecks from weak forecasting, procurement delays, unreliable supply chains, reimbursement constraints, misaligned provider incentives, and low levels of population awareness and trust.
- Policy reforms should be accompanied by system readiness assessments, aligned provider incentives, integrated digital tools, and sustained public engagement to build trust and improve uptake.

## Introduction

Ensuring continued access to affordable, quality medicines is central to universal health coverage (UHC) in low- and middle-income countries (LMICs) facing competing priorities and limited resources (Wirtz et al. 2017). This is particularly important for non-communicable diseases (NCDs), which require long-term, often lifelong, uninterrupted treatment alongside sustained service uptake. Global guidance recommends expanding benefit packages, regulating prices, promoting generic use, and strengthening procurement and supply chains (Bigdeli et al. 2017, World Health Organization 2019). Despite adopting these measures, many LMICs continue to experience persistent inequalities in medicine availability, affordability, and financial protection, with patients often facing high out-of-pocket spending, especially for long-term NCD treatment (Wirtz et al. 2017, Minghui et al. 2023). These challenges underscore the importance of a deeper understanding of how policies are implemented in practice across different complex health system contexts.

Ghana and Georgia offer timely cross-regional lessons on the health system determinants of access to NCD medicines. Both countries have pursued UHC-oriented reforms while adopting different approaches to improve pharmaceutical coverage (Seiter et al. 2025, Tsuladze et al. 2025). In Ghana, access to medicine reforms have largely operated within the National Health Insurance Scheme (NHIS), public-sector procurement arrangements, and framework contracting for public health commodities. In Georgia, access to medicine was expanded through state-funded medicine programmes (similar to Ghana) and later moved towards a pharmacy-based reimbursement model. While these reforms align with WHO recommendations (World Health Organization 2019), real-world implementation has been constrained by forecasting difficulties, procurement delays, supply chain distribution challenges, misaligned provider incentives, and limited public trust. This commentary highlights these four interrelated health systems issues that have impeded the implementation of access-to-medicines reforms in Ghana and Georgia. We draw on published peer-reviewed literature, WHO guidelines, and country-specific policy and audit reports. These sources were used to identify common implementation constraints affecting policy implementation for NCD medicines across the two health system contexts.

## Health systems constraints affecting access to non-communicable disease medicines

### System capacity and readiness

Ghana and Georgia adopted widely recommended centralized procurement to strengthen bargaining power and secure lower prices (World Health Organization 2020). However, both encountered challenges.

In Ghana, procurement responsibilities are split across the Ministry of Health, Ghana Health Service, and Teaching Hospitals. While framework contracts and centralized tenders aimed to stabilize supply (Koduah et al. 2023), supply chain assessments revealed large forecasting errors, frequent stockouts, and delays. Macroeconomic instability, currency fluctuations, inaccurate quantification, and delayed supplier payments prevented the timely delivery of medicines (USAID 2023). Consequently, many facilities resorted to ad hoc, costly procurement from private wholesalers, often disrupting continuity of care (Ashigbie et al. 2016).

Georgia faced similar constraints during initial centralized procurement for NCD medicines (State Audit Office of Georgia 2018). Limited technical capacity in forecasting and logistics planning hindered effective implementation, and distribution difficulties resulted in stockouts and geographical inequities for NCD medicines. Although the oncology programme performed better, the broader chronic medicines initiative saw low uptake and eroded public confidence (Tsuladze et al. 2025).

Global guidance promotes centralized procurement and pooled purchasing (World Health Organization 2020). Yet, effective centralized procurement depends on strong supply chain infrastructure, including reliable information systems, skilled personnel, warehousing and transport capacity, and sound financial management. In contexts where these components are weak, centralization can exacerbate existing bottlenecks. Countries with constrained public-sector logistics capacity may therefore consider alternative or hybrid approaches, such as outsourcing selected functions to non-public organizations.

### Provider capacity, incentives, and behaviours

Provider decisions—on prescriptions, stocking, patient prioritizations, and charging additional fees—can determine how policy reforms affect access to medicines.

In Ghana, low reimbursement rates, delayed payments, and frequent stockouts created disincentives for providers (USAID 2023), who introduced informal fees to cover financing gaps or prescribed higher-priced branded alternatives instead of reimbursed generics. These practices undermined financial protection and disrupted continuity of NCD treatment (USAID 2023).

In Georgia, early reimbursement caps placed financial burdens on patients whose annual medicine needs exceeded programme limits (Gorgodze et al. 2025, Tsuladze et al. 2025). Inadequate engagement and training of primary care providers meant that many did not systematically inform patients about UHC benefits or align prescribing practices with programme requirements. Some providers preferred non-covered brands due to perceptions of better quality or incentives from pharmaceutical suppliers (Tsuladze et al. 2025).

Global policy promotes generic substitution or copayment structures (Wirtz et al. 2017, World Health Organization 2019, Minghui et al. 2023), but the Ghana and Georgia cases demonstrate the importance of behavioural dynamics. Provider incentives, administrative burdens, reimbursement rules, and reliability of supply all shape whether providers view reforms as workable and legitimate. Improving access to medicines, therefore, requires targeted attention to provider behaviour, aligning incentives, and ensuring accountability.

### Digital tools and data use

Both Ghana and Georgia have invested in digital technologies for medicines management. Logistics Management Information System platforms track stock levels and inform procurement, while electronic prescribing and electronic health records aim to strengthen clinical decision-making and monitor prescribing patterns.

In Ghana, digital systems have improved visibility across the supply chain (Seiter et al. 2025), but persistent gaps in data completeness and timeliness limit their usefulness for decision-making. Implementation of the national electronic pharmacy platform and e-prescribing has progressed slowly in rural and primary-level facilities (Seiter et al. 2025).

In Georgia, adoption of mandatory electronic prescribing has been inconsistent (State Audit Office of Georgia 2018). Private insurers and urban facilities adopted digital tools more rapidly than others, resulting in fragmented data. Limited use of e-prescribing affected the country’s ability to monitor prescribing behaviours, identify irrational use, adjust reimbursement policies, or direct resources to underserved areas (State Audit Office of Georgia 2025).

These experiences demonstrate that digitalization requires investments in infrastructure, workforce capacity, supportive supervision, and mechanisms that encourage data use. Without these, digital platforms can add an administrative burden without meaningful improvements. For LMICs, digital systems can become powerful enablers of access, but only when introduced with realistic expectations and integrated into a system-strengthening strategy.

### Public awareness and trust

Public trust influences the success of reforms. In Georgia, communication during the rollout of the chronic medicines programme was limited, and primary care providers were not fully engaged as trusted messengers (Tsuladze et al. 2025). Surveys revealed that most people didn’t understand programme benefits, thus constraining enrolment (State Audit Office of Georgia 2018). Perceptions of government-funded medicines being inferior contributed to low uptake and influenced prescribing patterns. Supply disruptions and administrative barriers further eroded public trust (Tsuladze et al. 2025).

In Ghana, although the NHIS is widely praised (Arthur 2025), a limited understanding of entitlements restricted community involvement in decision-making, and persistent illegal fees continuously undermined access and fuelled unmet expectations (Aboagye et al. 2021).

Across both countries, limited investment in community-level communication and engagement with civil society, patient groups, and local leaders hindered public awareness and trust. Reforms cannot succeed without strong communication and social legitimacy. Benefit packages that are poorly understood or perceived as low quality will experience limited uptake, regardless of generosity. Effective policies, therefore, require ongoing, well-resourced communication and proactive community engagement.

## Implications for policy and research

1. System readiness assessments are critical to guide context-appropriate reforms.
2. Provider incentives and behaviours can improve ownership, compliance, and the effectiveness of reforms.
3. Integrating digital tools with broader governance and accountability mechanisms can strengthen rather than complicate existing systems.
4. Public awareness and trust are core components of policy design and implementation through sustained outreach and communication.
5. Comparative, cross-regional research can deepen understanding of implementation dynamics across different health system contexts.

## Conclusion

Improving access to NCD medicines requires an in-depth understanding of complex health systems contexts. The effectiveness of centralized procurement, price regulation, and expanded benefits is shaped by system capacity, provider behaviour, digital infrastructure, and public trust. As LMICs pursue UHC goals, policymakers and global actors must shift from focusing solely on policy adoption to addressing the practical realities of implementation in complex, resource-constrained systems.

## Data Availability

No new data was generated.

## Reflexivity statement

The authors of this study comprise three women and five men, representing diverse levels of seniority and geographical backgrounds. Their professional and public service experiences span multiple sectors, and they collectively provide mentorship to early- and mid-career researchers. The authors are based in Ghana, Georgia, and the United Kingdom.
